# Initial results with [^18^F]FAPI-74 PET/CT in idiopathic pulmonary fibrosis

**DOI:** 10.1007/s00259-023-06564-y

**Published:** 2023-12-20

**Authors:** Yuriko Mori, Vasko Kramer, Emil Novruzov, Eduards Mamlins, Manuel Röhrich, René Fernández, Horacio Amaral, Cristian Soza-Ried, Barbara Monje, Eduardo Sabbagh, Matías Florenzano, Frederik L. Giesel, Álvaro Undurraga

**Affiliations:** 1 Department of Nuclear Medicine, Medical Faculty and University Hospital Duesseldorf, Heinrich-Heine-University Duesseldorf, Moorenstrasse 5, 40225 Duesseldorf, Germany; 2 Center for Nuclear Medicine and PET/CT, PositronMed, 7501068 Providencia, Santiago Chile; 3 Positronpharma SA, 7500921 Providencia, Santiago Chile; 4 Department of Nuclear Medicine, Heidelberg University Hospital, INF 400, 69120 Heidelberg, Germany; 5 Department of Nuclear Medicine, Mainz University Hospital, Langenbeckstraße 1, 55131 Mainz, Germany; 6 Instituto Nacional del Tórax, Santiago, Chile; 7 Clínica Universidad de los Andes, Santiago, Chile; 8 Institute for Radiation Sciences, Osaka University, Osaka, Japan; 9 Universidad de Chile, Santiago, Chile

**Keywords:** Fibroblast activation protein, FAPI, PET, Idiopathic pulmonary disease, Fibrosis

## Abstract

**Abstract:**

Idiopathic pulmonary fibrosis (IPF) is a chronic fibrosing interstitial lung disease with a poor prognosis. ^68^Ga-labeled FAP ligands exhibited highly promising results due to the crucial role of activated fibroblasts in fibrosis imaging of the lung. However, ^18^F-labeled FAP ligands might provide qualitatively much higher imaging results with accompanying economic benefits due to large-scale production. Thus, we sought to investigate the potential of [^18^F]FAPI-74 prospectively in a small patient cohort.

**Methods:**

Eight patients underwent both [^18^F]FAPI-74-PET/CT and HRCT scans and were then compared with a control group without any fibrosing pulmonary disease. The tracer uptake of fibrotic lung areas was analyzed in synopsis with radiological and clinical parameters.

**Results:**

We observed a positive correlation between the fibrotic active volume, the Hounsfield scale, as well as the vital and diffusing capacity of the lung.

**Conclusion:**

The initial results confirm our assumption that [^18^F]FAPI-74 offers a viable non-invasive assessment method for pulmonary fibrotic changes in patients with IPF.

## Introduction

Idiopathic pulmonary fibrosis (IPF) is a chronic fibrosing interstitial lung disease of unknown cause with a very poor prognosis that is characterized by abnormal collagen accumulation in the lung parenchyma, leading to progressive impairment of regeneration and repair [1, 2]. Various genetic and environmental factors have been considered to trigger the cascade of inflammatory events, leading to permanent loss of normal lung tissue [3]. High-resolution computed tomography (HRCT) and pulmonary function tests represent currently the mainstay of diagnosis, of which HRCT is considered a gold standard due to the reported positive predictive value of 90% [4, 5]. However, since these examinations reveal fibrotic changes rather late during the course of the disease, lung areas with minor fibrotic changes in the short term during therapy might be difficult to detect—thus impairing the clinical decision-making process with optimal dynamic disease management.

The introduction of fibroblast activation protein (FAP) ligands as fibrosis monitoring agents offers great potential in order to fulfill this unmet clinical need. Several studies investigating the efficacy of ^68^Ga-labelled FAP ligands provide highly promising insights into the mechanism and clinical course of fibrosis in lung parenchyma [6–10]. However, due to the well-known drawbacks of ^68^Ga-labelling such as high radiation burden with suboptimal imaging quality and poor cost-effectiveness, ^18^F-labelled FAP ligands draw great attention in routine clinical care [11, 12]. To the best of our knowledge, this is the first study, analyzing the efficacy of [^18^F]FAPI-74-PET/CT prospectively in a small patient cohort.

## Material and methods

### Patients

Eight patients suffering from IPF were recruited at the National Thorax Institute, Santiago (Chile) between March and July 2021. IPF was diagnosed based on clinical parameters, functional tests (spirometry and DLCO), the radiologic pattern on CT, and pathology results in some cases, assessed by an experienced pulmonology physician in conjunction with a radiologist, both with over 20 years of experience in the interpretation of lung disease. The cohort included 4 males and 4 females with a median age of 71 years (range 66–77) (Table 1). The time since initial diagnosis ranges from 5–10 years. The control group included six male patients who had undergone [^18^F]FAPI-74 within the diagnostic workup of a cancer entity (pancreas cancer *n* = 2; rectum cancer *n* = 1, sarcoma *n* = 3) with no evidence for a clinical or radiological sign of pathologic pulmonary finding between March and June 2021 (median 70 years, range 68–78). The study was approved by the regional ethics committee board (CEC SSM Oriente/05012021) and conducted in accordance with the Declaration of Helsinki, Good Clinical Practices, and national regulations. Written informed consent was obtained from all patients before undergoing any intervention and on an individual basis. The imaging and clinical data were then anonymized and analyzed retrospectively at the University Hospital of Duesseldorf (UKD).

**Table 1:** Patient characteristics

Patient no	Age (y)	[^18^F]FAPI-74 (MBq)	FVC (%)	DLCO (%)
1	67	248	88	63
2	77	285	59	37
3	72	266	85	56
4	76	281	62	64
5	76	281	111	44
6	70	259	84	53
7	66	244	80	64
8	69	255	82	80

*FVC* forced vital capacity, *DLCO* diffusing capacity for carbon monoxide, *CO* carbon monoxide

### Image acquisition

All PET scans were performed 60 min after intravenous tracer administration using a Biograph mCT Flow scanner (Siemens, Erlangen, Germany). Imaging data were acquired in 3-dimensional mode (matrix, 220 × 220) with an acquisition time of 3 min per bed position. Attenuation correction was performed using CT data (170 mAs, 100 kV, 2-mm slice thickness). The injected activity of [^18^F]FAPI-74 was 199–239 MBq.

### Image analysis

Tracer uptake in the pulmonary fibrotic lesions was quantified with mean and maximum standardized uptake values (SUV_mean_ and SUV_max_) and the fibrotic active volume (FAV). FAV was determined in volumes of interest (VOI) with isocontour set at 45% of the maximum tracer uptake within the respective region of interest (ROI) using the automated lung segmentation protocol of a dedicated software package (Hermes Hybrid Viewer, Affinity 1.1.4; Hermes Medical Solutions, Stockholm, Sweden). Hounsfield scale (HU) in the corresponding area of FAV was quantified as HUmean and HUmax.

### Statistical analysis

Statistical analysis was performed using SigmaStat Version 3.5 (Systat Software, Inc., San Jose, CA, USA) and SigmaPlot Version 11.0 (Systat Software, Inc., San Jose, CA, USA) for graphical visualization. The comparison of tracer uptake between IPF and the control group was determined using a two-sided *t*-test. A *p*-value of less than 0.05 was considered statistically significant. The correlation between tracer uptake and clinical parameters was determined using Pearson’s correlation analysis.

## Results

### Tracer uptake in the fibrotic lung

**Fig.1:**
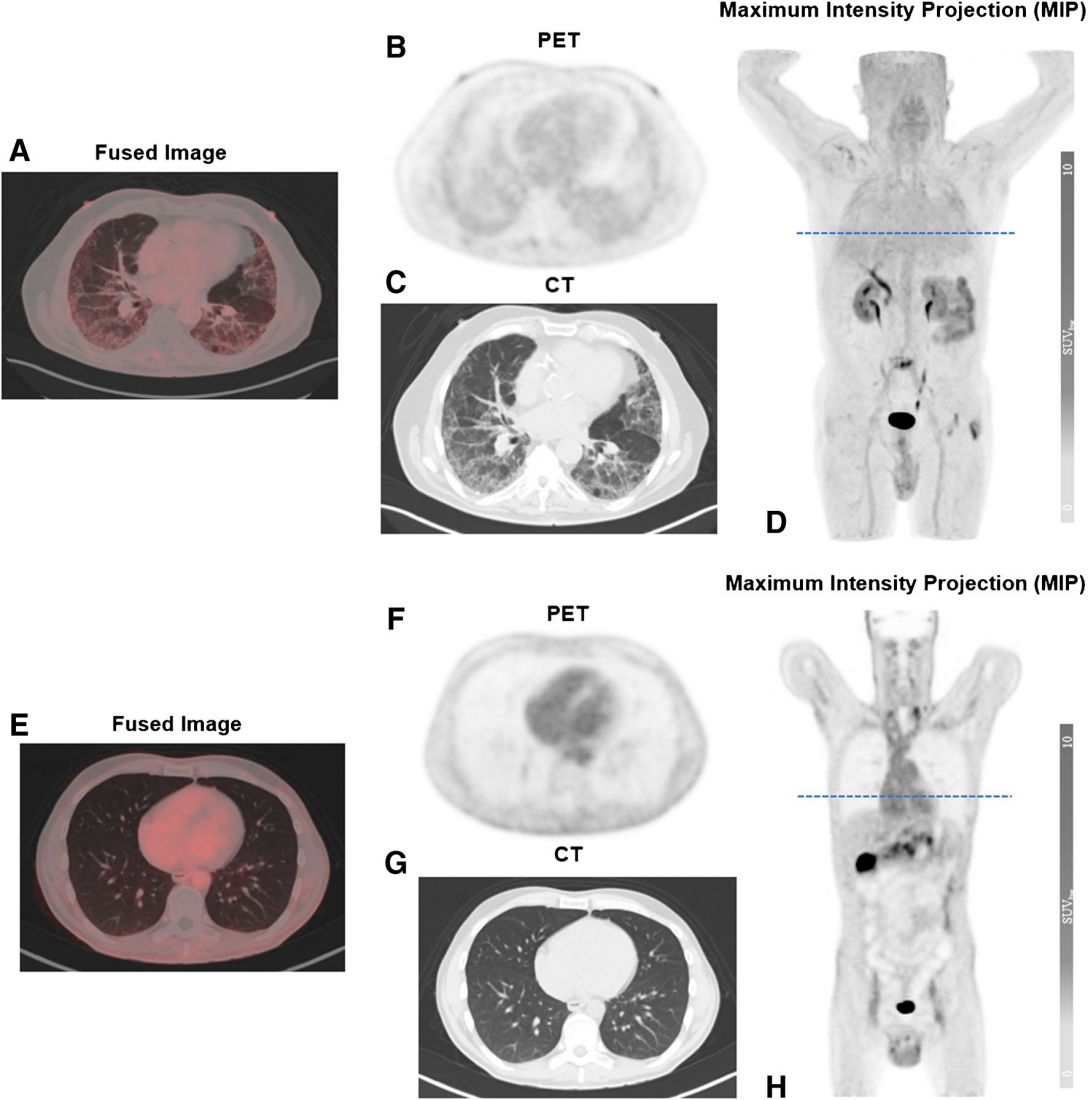
Positive [^18^F]FAPI-74 uptake in fibrotic lung tissue. Representative image of [^18^F]FAPI-74-PET/CT scan in a 68-year-old male patient with idiopathic pulmonary fibrosis in axial fusion (**A**), PET (**B**), CT (**C**) and maximum intensity projection (**D**). Positive tracer uptake is present in concordant regions in radiographic findings, but not in the non-fibrotic areas. Corresponding images of a 56-year-old male patient with pancreatic adenocarcinoma without any known lung disorder as control (**E**–**H**)

**Fig. 2:**
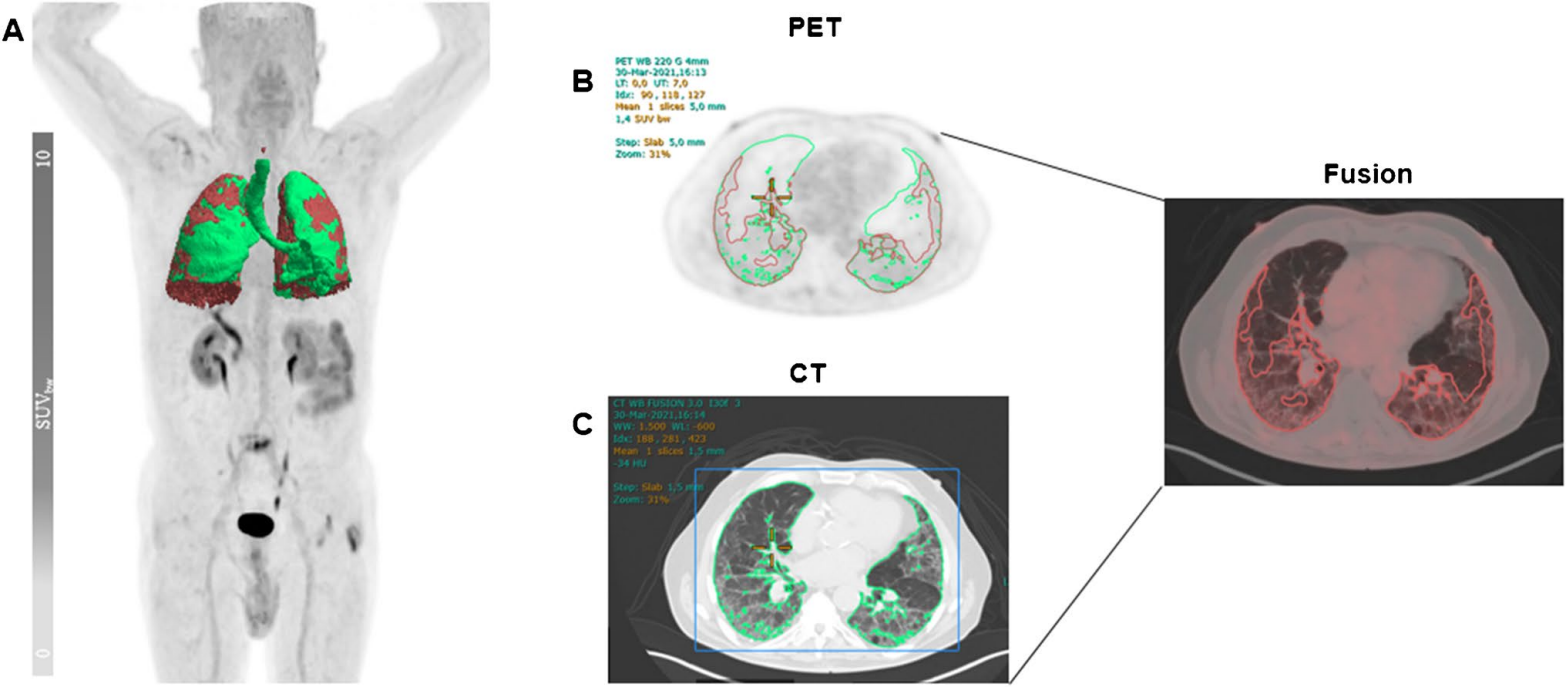
[^18^F]FAPI-74 uptake in FAV of fibrotic lung. Representative image of [^18^F]FAPI-74-PET/CT scan in a 76-year-old male patient with idiopathic pulmonary fibrosis with marked region of fibrotic active volume (FAV) (**A**), maximum intensity projection (**B**), PET (**C**), CT and (**D**) fusion

**Fig. 3:**
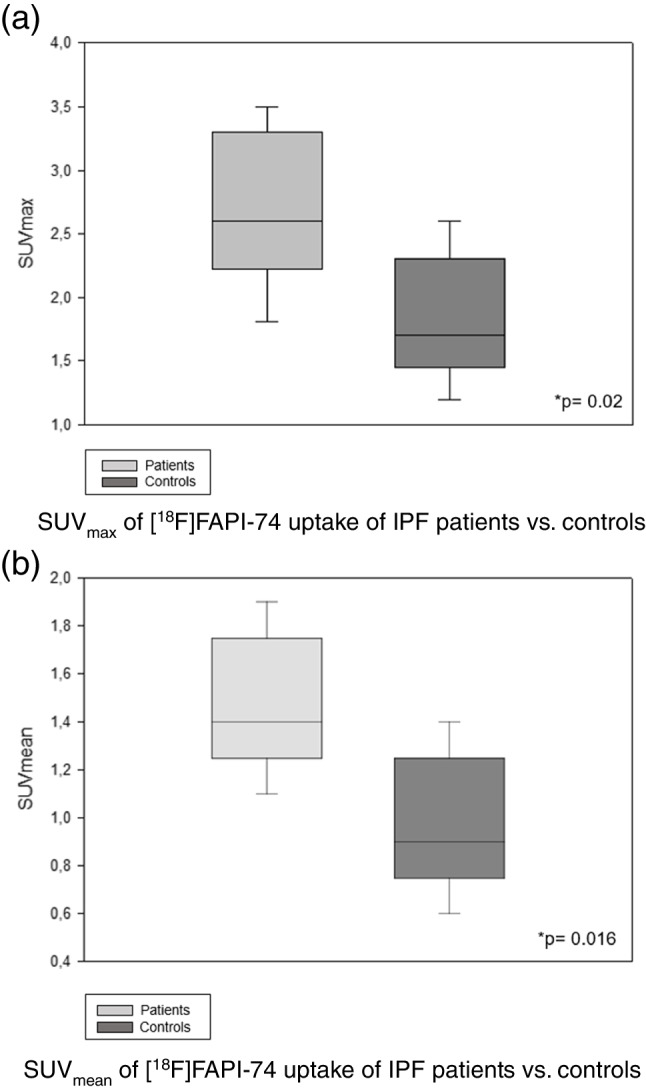
[^18^F]FAPI-74 uptake in patients with IPF. SUV data obtained in the patients and in controls are displayed as Whisker plots. **a** SUV_max_ of [^18^F]FAPI-74 uptake of IPF patients vs controls. **b** SUV_mean_ of [^18^F]FAPI-74 uptake of IPF patients vs. controls

Fibrotic changes in pulmonary parenchyma showed markedly elevated [^18^F]FAPI-74 uptake in the visual analysis (Fig. 1). FAV could delineate clearly the fibrotic changes in lung parenchyma (Fig. 2) with corresponding statistically significant, higher levels of SUV_max_ and SUV_mean_ in contrast to the control group (Fig. 3a and b).

### Correlation between tracer uptake and radiological and clinical parameters

**Fig. 4:**
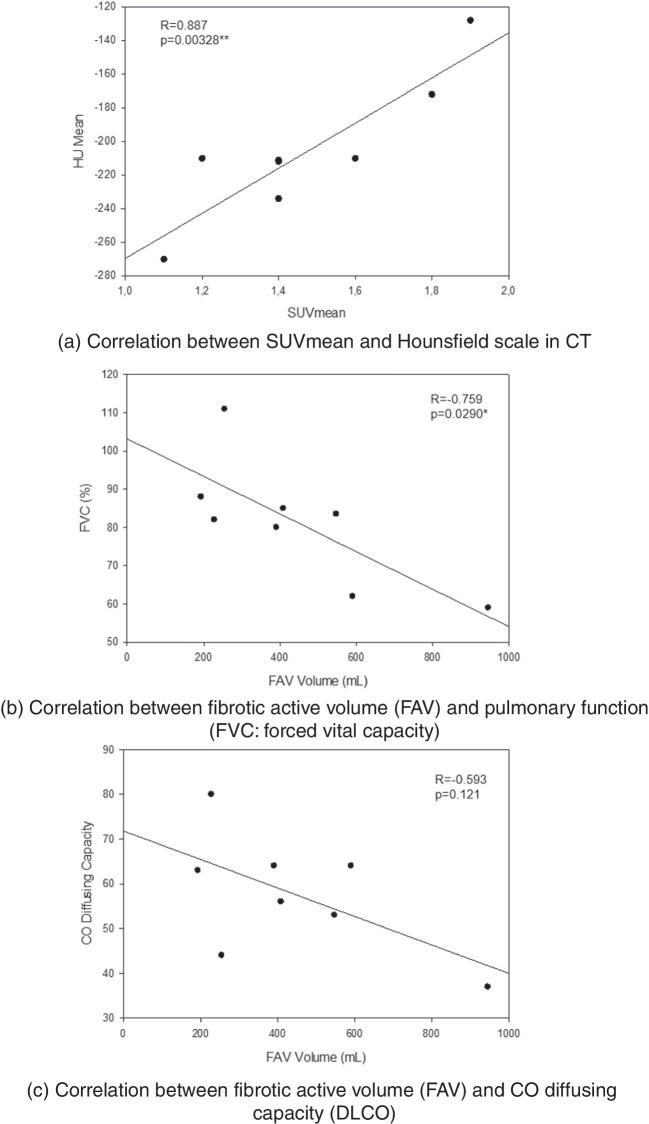
Correlation between [^18^F]FAPI-74 uptake and radiological and clinical parameters. **a** Correlation between SUV_mean_ and Hounsfield scale in CT. **b** Correlation between fibrotic active volume (FAV) and pulmonary function (FVC, forced vital capacity). **c** Correlation between fibrotic active volume (FAV) and CO diffusing capacity (DLCO)

We analyzed the correlation of SUV_mean_ with Hounsfield scale (HU) obtained from CT data in the corresponding area of FAV, which showed a significant correlation (*R* = 0.887, *p* < 0.005) (Fig. 4a). In addition, FAV showed a significant negative correlation to the forced vital capacity (FVC) (*R* =  − 0.759, *p* < 0.05), and a mild but not significant positive correlation to CO diffusing capacity (*R* =  − 0.593, *p* = 0.121) (Fig. 4b and c).

## Discussion

IPF is a relatively rapid-progressing lethal disease that is characterized by the abnormal accumulation of collagen in lung tissue, impairing its ability for repair and regeneration [1]. These changes in the extracellular matrix enhance the migration and activation of quiescent fibroblasts, which further accelerate the fibrosing process [2]. Thus, early detection and initiation of antifibrotic therapy as well as a reliable therapy-monitoring tool appear to be essential for therapy management. Based on this unmet clinical need, several research groups investigated the novel-tracer family, FAP ligands, in the assessment of fibrosing processes of the lung with highly interesting, promising results [6–9].

In the light of encouraging results obtained with ^68^Ga-labeled FAPI [13–15] and to overcome some drawbacks of ^68^ Ga-labeling, we hypothesized that the quantification of lung fibrosis using fibrotic active volume in [^18^F]FAPI-74 might correlate with the clinical severity and thus could serve as a non-invasive evaluation method. To the best of our knowledge, this is the first study to investigate [^18^F]FAPI-74 in pulmonary fibrosis.

In the current study, we could demonstrate that patients with clinically impaired lung function showed higher fibrotic active volume with corresponding pulmonary area with positive FAPI uptake. Because FAP tracers are known to accumulate in the non-quiescent, activated fibroblasts, our results indicate that FAPI-PET/CT can quantify ongoing tissue remodeling, which might predict disease progression. [^18^F]-FAPI uptake (median SUV_mean_) in our study was 1.40 (1.10–1.90) in IPF vs. 0.90 (0.60–1.40) in the control group, similar to the results of Bergmann et al. [7] using [^68^Ga]-FAPI-04 with median SUV_mean_ 0.80 (0.60–2.10) in ILD vs. 0.50 (0.40–0.50) in controls. A slightly higher tracer uptake observed in our study might result from the longer half-life of ^18^F remaining in the region of interest at the time point of the PET scan. Interestingly, Bergmann et al. [7] have previously shown in the mentioned prospective study in patients with interstitial lung disease (ILD) in systemic sclerosis that [^68^Ga]-FAPI-04 accumulation at baseline was associated with the risk for ILD progression in the follow-up period of 6–10 months. The authors observed this disease progression independently of other known risk factors, such as the extent of involvement in HRCT at baseline and FVC at baseline. Considering this, our data as a one-point-assessment might imply further prognostic relevant information, which should be verified in the long-term period.

We found the fibrotic active volume of the fibrotic lung significantly correlates with the Hounsfield scale on CT. This result is consistent with the previous work of Röhrich et al. [8], which demonstrated that the tracer uptake of [^68^Ga]-FAPI-46 correlates with radiographic parameters such as fibrosis (FIB)- and ground-glass opacity (GGO) index. Bergmann et al. [7] also reported on minor correlation of [^68^Ga]-FAPI-04 with the extent of ground glass opacities. Because radiographic signs like ground-glass opacity can be easily affected by morphological changes of other causes (e.g., alveolitis or edema of infectious backgrounds) and might be rather unspecific, these findings further support the utility of FAPI-PET/CT in the clinical setting, as it contains the radiographic as well as the fibrotic information, which might allow insight into the current disease activity with possible prognostic implications. Regarding the general image quality, ^18^F-labeled compounds reveal higher spatial resolution allowing the detection of smaller lesions as compared to ^68^Ga-labeled compounds, combined with several other advantages including cost-effective production and centralized supply. Whereas ^18^F-labeled compounds allow larger batch production in facilities equipped with cyclotrons at lower cost and can be delivered to remote centers (satellite concepts), the typical batch size of a ^68^Ge/Ga^68^ generator allows the daily clinical performance of only a few patients per elution and requires an on-site and on-time synthesis of the radiotracer, also because of the shorter half-life (68 min).

Further, we could demonstrate an investigator-independent, readily reproducible evaluation method using a dedicated software package (Hermes Hybrid Viewer, Affinity 1.1.4; Hermes Medical Solutions, Stockholm, Sweden) that provides a clear delineation of fibrotic or chronic inflammatory changes of lung parenchyma in terms of fibrotic active volume. Although fibrotic active volume, as already mentioned, displayed a statistically significant, negative correlation with forced vital capacity, the correlation with CO-diffusing capacity could not reach statistical significance, most probably due to the small cohort size.

The main limitation of our study seems to be the small cohort size. Additionally, PET images were acquired without respiratory gating so that there might be some difference in tracer uptake between respiratory phases.

## Conclusion

These encouraging results pave the way for further, multicentric, large-scale trials for the evaluation of fibrosing processes in lung parenchyma with [^18^F]FAPI-74.

## Data Availability

The data used and/or analyzed during the current study are available from the corresponding author upon reasonable request.
